# Catalytic Fast Pyrolysis of Poly (Ethylene Terephthalate) (PET) with Zeolite and Nickel Chloride

**DOI:** 10.3390/polym12030705

**Published:** 2020-03-23

**Authors:** Hang Jia, Haoxi Ben, Ying Luo, Rui Wang

**Affiliations:** 1 Key Laboratory of Energy Thermal Conversion and Control of Ministry of Education, Southeast University, Nanjing 210096, China; 18707126420@163.com (H.J.); danteli_an2410@163.com (Y.L.); wr3502140301@163.com (R.W.); 2 School of Energy and Environment, Southeast University, Nanjing 210096, China

**Keywords:** poly (ethylene terephthalate) (PET), pyrolysis, zeolite, NiCl_2_, Fourier transform infrared spectroscopy (FT-IR), ^13^C nuclear magnetic resonance (NMR), pyrolysis mechanism

## Abstract

The pyrolysis of poly (ethylene terephthalate) (PET) in the presence of ZSM-5 zeolite and NiCl_2_ as a catalyst was studied at different temperatures under N_2_ atmosphere. Quantitative ^13^C nuclear magnetic resonance (NMR) and Fourier transform infrared spectroscopy (FT-IR) were applied to characterize the waxy and solid residue. The carboxyl and aliphatic hydroxyl groups in the waxy residue have been greatly depleted after the use of zeolite during pyrolysis on the basis of the results of ^13^C NMR and FT-IR analysis. The proportion of aromatic hydroxyl groups increased by 21.82% when the mass ratio of zeolite to PET was set to 2.0/1.0. The results indicate that ZSM-5 is able to facilitate the decomposition of carboxyl, aliphatic groups, and ether bonds in the primary products produced from the pyrolysis of PET. In addition, the deoxygenation effects on the waxy products have been significantly enhanced with the addition of zeolite based on the results of NMR.

## 1. Introduction

Polyethylene terephthalate (PET) is one of the most widely used polymers in packaging, electronics, storage, and personal care products across the world. Then, the extensive use of PET led to the growth of PET waste accumulation. For example, according to data from the European Union, in 2018, 29.1 million tons of plastic waste including PET were generated; only approximately 32.5% was recycled and 42.6% was recovered through energy recovery techniques. Regrettably, 27.3% went to landfills [1]. However, plastics take up to hundreds of years to be biodegraded naturally, and they currently occupy a large volume of costly landfill space, which causes serious environmental problems. However, plastics waste recycling has increased almost 80% in the last 10 years [1], which is similar to the packaging recycling (74%). Since PET accounts for a large proportion of the packaging materials, it is vital to explore more reliable and sustainable methods to recycle PET waste.

Thermochemical conversion is a promising route to produce clean energy and chemical raw materials from these waste plastics while diverting them from landfills [2,3]. It is an efficient way to degrade polymerized molecules into less complex ones by heating in a space filled with inert gas. The pyrolysis of PET is regarded as the most desirable method to convert PET waste into fuels and useful chemical products [4,5,6]. Among them, pyrolysis oils are the primary products; however, these have several challenging properties including a mixture of compounds with a high oxygen content, poor volatility, and high acidity and viscosity due to the presence of benzoic acid and terephthalic acid and so on [7,8,9,10], which significantly limits its usage as fuels or chemicals. Hence, catalytic pyrolysis seems to be a feasible and indispensable method to solve this problem due to its decarboxylation or decarbonylation ability for pyrolytic vapor.

Many researchers have examined the influence of kinds of catalysts such as zeolite and metal salts to increase the field of pyrolysis oils and upgrade the properties of liquid products during the pyrolysis of PET. For instance, Kumagai et al. used a vertical tube reactor to pyrolyze different kinds of plastics including PET at 600 and 700 °C in the presence and absence of either CaO and or Ca(OH)_2_. They concluded that the production of sublimating substances during the pyrolysis of PET was significantly reduced after the addition of CaO [11]. Furthermore, Masuda et al. developed a new method to decompose terephthalic acid produced from the pyrolysis of PET into useful liquid hydrocarbons, using inexpensive catalysts such as transition metal oxides, including FeOOH, Fe_2_O_3_, Ni(OH)_2_, and NiO. They compared the effects of the above catalysts on the decomposition of terephthalic acid and found that FeOOH showed high activity, yielding no sublimate substances among the metal oxides [12]. Du et al. investigated ZSM-5 zeolite, CaO as catalysts in the pyrolysis of PET-based waste carpet at different temperatures and using different pyrolysis methods and analyzed the gas and liquid products by gas chromatography mass spectrometry (GC-MS). They studied different heating rates, thermal, and catalytic pyrolysis, with/without the co-feeding of steam and indicated that the conversion from PET to aromatic hydrocarbons was favored at higher temperatures and that slow heating rates can facilitate the production of benzene. They also found that ZSM-5 zeolite and CaO are both effective catalysts to completely deoxygenate the pyrolytic products [13]. A tandem micro-pyrolyzer was used to study the pyrolytic vapor produced from the pyrolysis of different types of plastics including polyethylene (PE), polypropylene (PP), polystyrene (PS), and PET using H-ZSM-5 as catalyst from 300 to 800 °C by Xue et al. [14]. They found that the decomposition of PET was reduced, but the formation of aromatic hydrocarbons was promoted compared to the results of pyrolysis without catalyst. Catalytically co-pyrolyzing PE and PET was also investigated and indicated that the yield of aromatic was increased but the yield of solid residue was reduced. Miandad et al. used modified natural zeolite (NZ), thermal activation zeolite (TA-NZ), and acid activation zeolite as catalysts in the pyrolysis of PET and other plastics at 600 °C and found that the optimal yield of pyrolysis oil (approximately 30%) was reached when the zeolite was activated by HNO_3._ Similar to the previous results, the analysis of pyrolysis oil was carried out by GC-MS and Fourier transform infrared spectroscopy (FT-IR) and indicated that the main components in the oil were aromatic compounds [15]. Al-asadi et al. pyrolyzed real PET-containing waste plastics in a horizontal tubular reactor with different Ni-loaded catalysts, including Ni/ZSM5, Ni/y-zeolite, Ni/beta-zeolite, and Ni/natural zeolite from 600 to 900 °C. They concluded that the highest yield of oil product was achieved in the presence of Ni/γ-zeolite and Ni/β-zeolite and oxygenates were significantly converted to non-oxygenated compounds in the oil products using Ni/ZSM-5 [16]. On the other hand, Laura S et al. chose sulfated zirconia (SZ) as catalyst in the pyrolysis of PET to recover benzoic acid products. They found that the recovery of benzoic acid achieved 26 wt % in the wax when the ratio of catalyst to PET increased up to 10 wt % [17].

In addition, compared to the massive research studies of other polymers such as PS, PE, and PP [18,19,20,21,22], the current research on PET is mainly focused on the pyrolysis of its mixture with other type of plastics or biomass. Sembiring et al. carried out the pyrolysis of the mixture of PP and PET with using a mixture of natural zeolite and bentonite as catalyst from 400 to 500 °C. They found that the proportion of each pyrolysis product was significantly influenced by temperature and catalyst, and the catalyst facilitated the further decomposition of solid products to produce more liquid oils. The addition of catalyst caused a dramatically increase of non-oxygenated aromatic hydrocarbons. Furthermore, consistent with the pyrolysis of PET, the main problem of the recovery of products from the pyrolysis of lignin was its high oxygen content and poor volatility. Hence, the idea of pyrolyzing lignin may be referenced [23]. Geng et al. used analytical pyrolysis methods (Py-GC-MS) to analyze the pyrolytic vapor obtained from the pyrolysis of alkali lignin with nickel formate. They concluded that the liquid yield was greatly improved using nickel formate as the additive and the contents of alkylphenols and aromatics in the pyrolysis oil were significantly promoted as well [24]. French et al. attempted to upgrade the pyrolysis vapors produced from lignin using zeolite as additives. They found that the optimal yield of hydrocarbons (approximately 16 wt %, including 3.5 wt % of toluene) was reached with the use of nickel, cobalt, iron, and gallium-substituted ZSM-5 zeolite [25]. Furthermore, it was reported that the selectivity of aromatic hydrocarbon can be influenced by the introduction of Ni^2+^ [26].

Driven by prior studies in PET or lignin pyrolysis, we found that most of the previous catalytic pyrolysis studies have focused on the effect of several parameters such as temperature, heating rate, different type of PET, and common catalysts on pyrolysis products and its distribution. Nevertheless, only a few studies investigated the synergistic effect of ZSM-5 zeolite, nickel chloride, and their dosage and temperature on the pyrolysis of PET. In view of the effective application of nickel salts and nickel-substituted zeolite in the pyrolysis of lignin coupled with the inexpensive price of nickel chloride, the work presented here focuses on the relationship between the structure of liquid products and the dosage of catalyst and temperature. Furthermore, different temperature and catalyst dosages have been studied to figure out the mechanism of PET pyrolysis. Based on the detailed structure information of pyrolytic products provided by ^13^C nuclear magnetic resonance (NMR) analysis, the change of functional groups in the liquid products can be easily found before and after using the catalyst [27].

## 2. Materials and Methods

### 2.1. Materials

**Figure 1:**
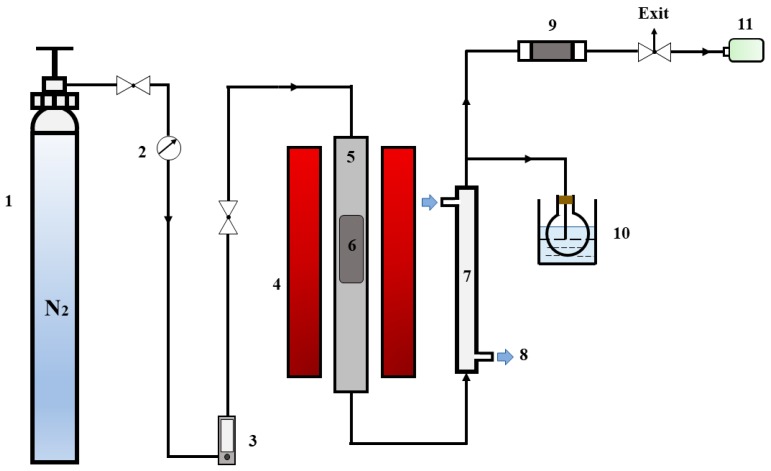
Schematic diagram of experimental apparatus. 1. N_2_ cylinder; 2. Pressure gauge; 3. Flow meter; 4. Tube furnace; 5. Quartz tube; 6. Quartz boat; 7. Condenser; 8. Cooling ethanol; 9. Quicklime dryer; 10. Ice-water trap; 11. Sampling bag.

PET pellets with three different molecular weights (Mw = 50,000 g/mol) were all purchased from HuaChuang Plastic Co., Guangdong, China. The purity of PET power exceeds 99%. The PET pellets (particle size: 3 mm) were grinded and sieved to gain the PET powder with the size of 100–150 μm. The characterization of PET plastics is shown in Table 1. Overall, the results of characterization are similar to that in the most recent literature [8,10,13]. PET had a high carbon content and relatively low hydrogen content due to the existence of aromatic rings, ester, and a carboxylic group as described in Figure 1. The C, H, and O composition of the sample was 61.87 wt %, 4.35 wt %, and 33.78 wt %, respectively, which is similar to the theoretical values for pure PET (C, 62.5 wt %; H, 4.2 wt %; O, 33.3 wt %). Proximate analysis was also carried out under reported condition [11] and was listed in Table 1. The contents of volatiles, fixed carbon, and ash of PET were 88.54%, 9.37%, and 2.09%, respectively, suggesting the formation of large amounts of oil or gas products during pyrolysis.

### 2.2. Catalyst Preparation

ZSM-5 zeolite (NKF-5, SiO_2_/Al_2_O_3_ = 46:1) was purchased from Nankai University Catalyst Co., Ltd., Tianjin, China. The zeolite was calcined at 550 °C for 6 h in the quartz tube under inert atmosphere in order to avoid water absorption of the hyperthermal zeolite during the cooling process before the experiment. The zeolite was immediately filled into a dry bottle for subsequent experiments when the inside temperature of quartz tube is near the room temperature.

NiCl_2_ catalyst was prepared by using nickel chloride hexahydrate as a precursor. The hydrate was ground to powders and then put into the dryer. The powders were dried to remove its own crystal water at 140 °C for 2 h. Meanwhile, the powders needed to be stirred manually to avoid clumping. The sign of complete removal of crystal water is that the color of powders changes from dark green to khaki yellow.

### 2.3. Tube Furnace Pyrolysis

The thermal and catalytic fast pyrolysis experiments of PET were carried out under N_2_ atmosphere in a horizontal tubular reactor (60 mm (ID) × 1000 mm (L)), and its schematic diagram is displayed in Figure 1. The quartz boat filled with PET sample and catalyst was first placed inside the quartz tube embedded in the tube groove inside the electric furnace. Then, the quartz tube was connected to the N_2_ cylinder through the gas pipe and continuously flushed with nitrogen at a flow rate of 200 mL/min for 10 min to eliminate any oxygen in the tube. The temperature inside the quartz tube reactor was measured by a K-type thermocouple connected to a temperature recorder, and the real-time of temperature and heating in the reactor during the operation can therefore be reflected effectively. The tube reactor loaded with the PET sample and catalyst was inserted into the preheated furnace immediately as soon as the temperature reached the predetermined value. After finishing the above operations, the quartz boat loaded with PET was heated up to a diverse final temperature (450, 600 °C), which needed to be held for 30 min to ensure that the pyrolysis is fully completed. The solid residue and the gas residence time was adjusted to 30 and 5 min respectively at an extremely fast heating rate. It can also contribute to the full decomposition of the volatiles released.

As is shown in Figure 1, the condenser was connected downstream of the quartz tube, which was linked with a freezing fluid circulation device, which used ethanol as frozen liquid inside, and the condensation temperature was set to −5 °C. The other outlet of the condenser is followed by a round-bottom flask immersed in an ice-salt bath (approximately 0–3 °C). The volatiles generated by the pyrolysis of PET were condensed in the condenser and flowed into the flask to be collected. Meanwhile, the incondensable gas was collected into the gas-sampling bag after passing through the dryer, and then the gas was discharged into the air after the treatment. Finally, the remaining products deposited in the quartz boat were collected and weighed to determine the yield as soon as the heating area cooled down to room temperature.

Two different pyrolysis products were collected at the end of every experiment: the pyrolysis char that remained in the quartz boat, the condensable waxy products collected inside the tube, and the connections between the tube and the condenser. The recovered waxy products mixed with pyrolysis oil and a small amount of solid powder accounting for only a fraction of all solid powder can only be dissolved in pyridine. Dhahak et al. tried to use other solvents such as water, ethanol, hot methanol, acetone and acetate, etc. to dissolve the waxy product and found that no other solvents work better than pyridine [28]. Therefore, the waxy product that adhered to the wall of the quartz tube needs to be washed with pyridine and followed by the evaporation of mixed liquid after each experiment. The majority of solid powder can be collected in the condenser and analyzed in the further study.

For each experiment, the quartz boat, the condenser, the connections, and other containers were all weighed before and after the pyrolysis. After that, the solid residue can be collected in the sample holder after the temperature drops to room temperature under N_2_ atmosphere, and the waxy products can also be collected through the above series of operation. The yield of solid residue and waxy products (including the waxy products that adhered to the wall of the quartz tube and connections and the rest of the solid powder produced during the pyrolysis of PET) were calculated by dividing the mass of the products by the initial mass of the sample. Meanwhile, the yield of the pyrolysis gas was calculated by difference. The repeatability of the experiments was confirmed by performing each thermal and catalytic fast pyrolysis experiment at least three times. The experimental standard deviation is reported in the experimental results in the form of error bars.

### 2.4. Analysis of Pyrolysis Products by FT-IR

The recovered solid power was made into samples by compression using KBr as the background and then was characterized by a Nicolet iS 10 Fourier transform infrared spectroscopy (FT-IR) spectroscopy purchased from Thermo Scientific Inc., Waltham, MA, USA. The spectra of FT-IR were obtained in the range of 3800 to 500 cm^−1^. The samples were analyzed repeatedly to acquire the most suitable one.

### 2.5. Analysis of the Waxy by ^13^C NMR

The waxy products produced from the pyrolysis of PET under different catalytic conditions were characterized by an AVANCE III HD 600 MHz Spectrometer purchased from Bryker Inc., Zurich, Switerland. The sample, which weighed 500 mg, was dissolved in 600 μL of deuterated dimethyl sulfoxide (DMSO-d6). The solvent peak appears as sevenfold peaks in ^13^C NMR spectra and the position of the peaks was at 39.52 ppm. In addition, the number of scans was 1024 and the pulse delay was set as 5 s [29,30]. The ^13^C NMR spectra were all processed in MestReNova v12.0 ((Mestrelab Research, Santiago De Compostela, Spain).

## 3. Results

**Figure 2:**
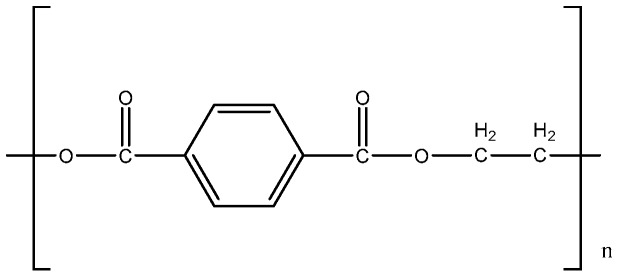
The structure of poly (ethylene terephthalate) (PET).

The structure of PET is described in Figure 2. According to the studies of Artetxe and Du, the random scission of the ester bonds leads to the formation of carboxyl groups and vinyl ester, etc. [8,13] when it is exposed to high temperatures (≥385 °C) [28]. Then, some smaller molecular bonds including acetaldehyde, CO_2_, CO, and ethylene were formed subsequently due to the instability of vinyl ester [31,32].

### 3.1. Effect of Temperature and Catalyst on the Products’ Distribution

**Figure 3:**
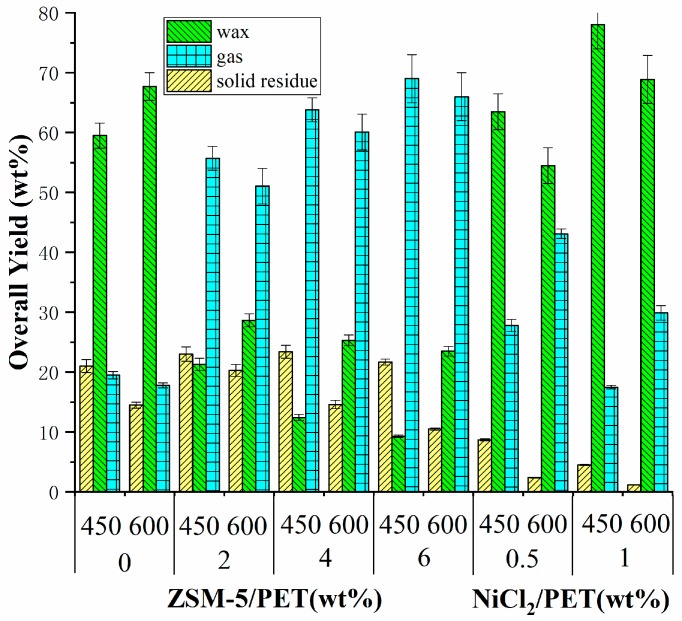
Yields of solid residue, the waxy products, and gas for the pyrolysis of PET with 0.0/1.0-6.0/1.0 (W_ZSM-5_/W_PET_) of ZSM-5 zeolite, 0.5/1.0-1.0/1.0 (W_NiCl__2_/W_PET_) of NiCl_2_ as catalyst at different temperatures (450 and 600 °C).

**Figure 4:**
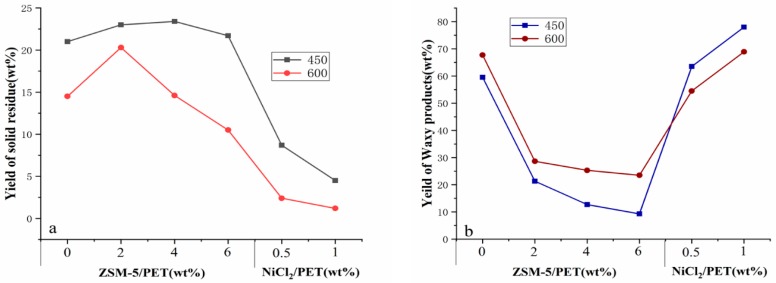
Effect of temperature and catalyst: PET mass ratio on the yield of solid residue (**a**) and waxy products (**b**).

Figure 3 shows the effect of the catalyst to samples mass ratio and the temperature on the overall yield of residue products, the waxy products, and the gas products. The tested operating conditions were set as follows: pyrolysis temperature from 450 to 600 °C, the residence time of pyrolytic vapors in the quartz tube (approximately 20 s) and different catalyst dosages from 0.5/1.0 to 6.0/1.0. As a result, the catalyst dosage had the biggest impact on the yield of products, followed by the pyrolysis temperature. The addition of a catalyst greatly changed the composition of the pyrolysis products. This is consistent with the results suggested in many previous studies. Generally, the effect of different catalyst formulation on pyrolysis products distribution was not the same. The ZSM-5 zeolite enhanced the production of the gas while reducing the production of the waxy products. In Figure 3, the yield of waxy products without any catalyst decreased from about 59.50 wt % and 67.70 wt % to below 10 wt % and about 23 wt % at different pyrolytic temperatures when the ZSM-5 was added as catalyst. This observation agreed well with the work by Du et al. [13], who carried out the catalytic fast pyrolysis of PET with a mass ratio of 20 at 600 °C and suggested that a sharp decrease (about 50.8%) of liquid products was found from 25 C% to 12.3 C% with the use of ZSM-5 as catalyst. On the contrary, the yield of gases increased from about 20 wt % to over 50 wt % on average, and it happened in almost all catalyst formulation. Laura et al. [17] used SZ as the catalyst during the pyrolysis of PET and found that the gas yield increased by 29% (from 38.19 wt % to 55.91 wt %) at 600 °C with the catalyst/PET mass ratio from 0 wt % to 10 wt %. This can be explained by the cracking of the C–C bond, leading to the formation of gas after using the catalyst [16]. The detailed products yield such as solid residue and waxy products are summarized in Figure 4a,b. Increasing the catalyst proportion did not have a clear effect on the yield of pyrolysis char, but it decreased the yield of the waxy product at 450 °C. As a result, the yield of the gases drastically increased. The yield of pyrolysis char was similar to the control group after the use of different dosages of zeolite, which indicated that the primary decomposition of PET was barely influenced by the zeolite. However, the situation based on the results of different nickel-supported zeolite catalyst such as Ni/ZSM-5, Ni/y-zeolite, etc. varies widely. Al-asadi et al. [16]. indicated that Ni/ZSM-5 and Ni/y-zeolite had the same influence on the formation of solid residue (decreased from 46 wt % to around 15 wt %), which agreed well with the results of our work, whereas a relatively high yield of solid residue was achieved using Ni/b-zeolite and Ni/natural zeolite. In addition, this finding does not apply to the conditions using nickel chloride as a catalyst. The yield of solid residue is much lower when applying nickel chloride as a catalyst compared to ZSM-5, whereas increasing the ratio of catalyst will also lead to a further decline in solid residue yield, which was consistent with a previous study by Geng et al. [24] who observed a significant decrease of solid residue (approximately 21%) and an obvious increase of liquid products (approximately 20%) during the pyrolysis of alkali using nickel formate. The addition of ZSM-5 zeolite led to the decrease of the waxy product during the pyrolysis. On the contrary, the yield of gas increased after the use of those two types of catalysts. When the temperature increased to 600 °C, the solid residue yield maximized when the waxy yield slumped at a catalyst/plastic mass ratio of 2. After this, the solid residue yield decreased with the catalyst (14.60 wt % at a ratio of 4 and 10.50 wt % at a ratio of 6). Based on the results relating to the yield of pyrolysis products, we can conclude that the secondary decomposition of volatile products was enhanced to produce more gas products at the expense of the waxy products with the use of ZSM-5 zeolite.

Based on the finding revealed in Figure 4a,b, the catalyst proportion was regarded to have a more profound effect on the PET pyrolysis at a higher temperature (600 °C). For instance, increasing the catalyst (ZSM-5) to PET mass ratio at 450 °C from 0 to 6 resulted in a 3.33% increase of the solid residue (from 21 wt % to 21.7 wt %) while at 600 °C, the solid residue decreased by 27.58% (from 14.5 wt % to 10.5 wt %). The same pattern applies for the use of NiCl_2_ as a catalyst for the pyrolysis of PET. The waxy yield increased by 26.42% (from 54.5 wt % to 68.9 wt %) at 600 °C, while it rose by 22.83% (from 63.5 wt % to 78 wt %) at 450 °C. This may indicate that the optimal temperature to activate both of two catalysts to promote the secondary decomposition is more than 450 °C, which is line with the study of Vouvoudi et al. [33].

### 3.2. Effect of Catalyst Dosage on the Wax Composition

The catalyst was covered on the top of the PET power in the quartz boat in our work. The residence time of pyrolytic volatile was basically determined by the catalyst proportion. Therefore, the effect of ZSM-5 and NiCl_2_ dosage was studied in the range of mass ratio from 0 to 6 and 01 respectively by adjusting the temperature at 600 °C and the nitrogen flow rate at a value of 200 mL/min.

#### 3.2.1. Analysis of Solid Powder by FT-IR

**Figure 5:**
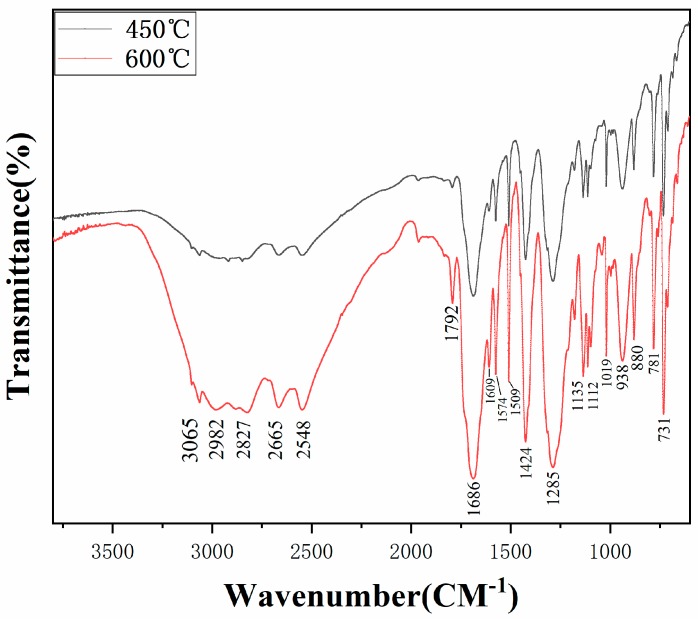
Fourier transform infrared spectroscopy (FT-IR) spectrums of the waxy products obtained at 450 °C and 600 °C without catalyst.

**Figure 6:**
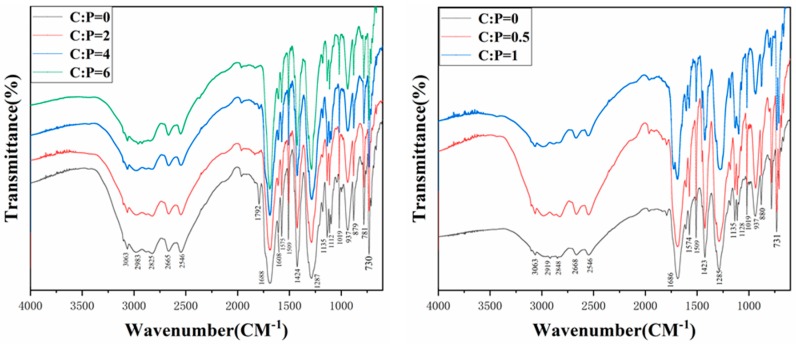
FT-IR spectrums of the wax collected at 600 °C with different (ZSM-5: **left**, NiCl_2_: **right**) catalysts.

Figure 5 shows the FT-IR analysis of the solid powder collected in a pipe at 450 °C and 600 °C without a catalyst with the gas flow rate of 200 mL/min. Based on the infrared spectrum, we can conclude that the temperature had a very limited effect on the functional groups composition of solid powder, which is inconsistent with the study of Laura et al. [17]. Figure 6 shows the spectra of solid products with different catalyst/PET mass ratios at 600 °C respectively. The spectrum of solid powder was distinguished by the peaks relating to olefin and carbonyl groups. Aromatic (C=C), C–H bands, and (=C–H) bands are situated at 1609 (1509), 3065, and 880 cm^−1^, respectively [34,35]. Two small but sharp peaks appear at 2982–2827 cm^−1^, indicating the presence of the C–H stretch in the alkenes, which is similar to the results of Dhahak et al. [28]. In addition, the strongest peak located at approximately 1685 cm^−1^ presents the C=O stretch and the existence of three strong peaks (at approximately 1260, 1085, and 1020 cm^−1^) referring to the ester bond (C–O) stretch reveals the existence of carboxylic acids.

These results suggest that the primary solid powders collected in the pipes and condenser are mainly formed by carboxylic acids and esters. The different spectrums at various temperatures and mass ratios of catalyst to plastic were compared with each other, indicating little difference among them. Hence, the results of the spectrum confirmed that PET solid products were mainly composed of aromatic acid. The FT-IR spectrum of solid powder during the pyrolysis of PET shown below did not to some extent agree well with the previous study [32]. A small olefin group (C=C) was found in the spectrum with/without catalyst, while around 60 vol.%, olefins were confirmed in the tar produced from the pyrolysis without any catalyst [31]. It may be explained by the different sources of solid powder and pyrolysis, which contains solid powder and other liquid products.

#### 3.2.2. Analysis of Waxy Products by Quantitative ^13^C NMR

An elaborate analysis was implemented via ^13^C NMR to characterize the change of functional groups in the waxy products. A detailed ^13^C NMR chemical shift assignment range database of components presented in the pyrolysis oil proposed by Ben et al. was applied for the analysis of the waxy products to facilitate this study. The mentioned chemical shift database is summarized in Table 2.

**Figure 7:**
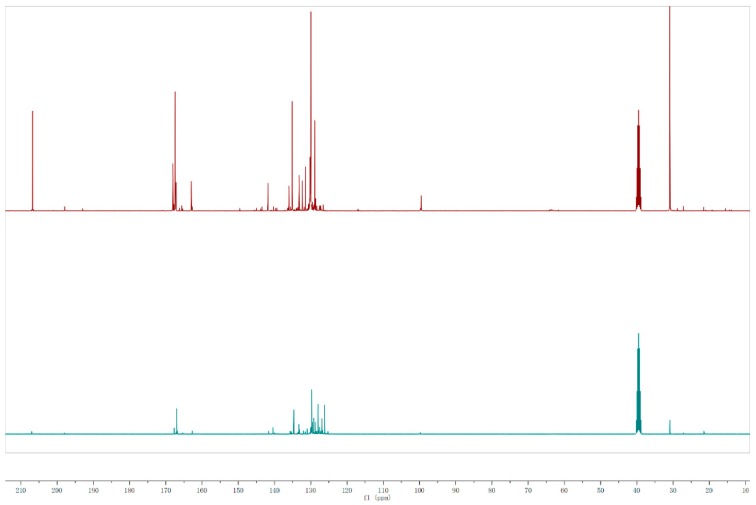
Quantitative ^13^C NMR spectra for the waxy products produced by the thermal pyrolysis of PET (**top**) and catalytic fast pyrolysis with ZSM-5(1:2) as a catalyst (**bottom**) at 600 °C for 30 min.

**Figure 8:**
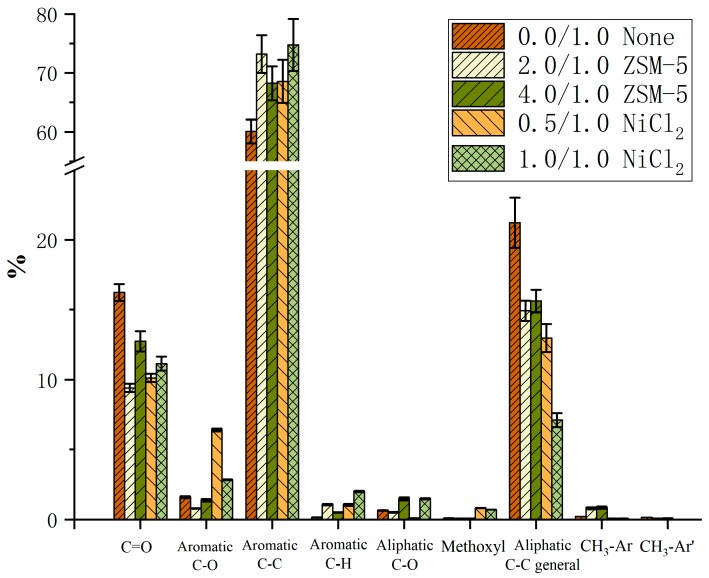
Integration results for the waxy products collected from the pyrolysis of PET powders with 0.0/1.0 (without catalyst), 2.0/1.0 (W_ZSM-5_/W_PET_), 4.0/1.0 (W_ZSM-5_/W_PET_), and 0.5/1.0 (WNiCl_2_/W_PET_) of NiCl_2_ at 600 °C for 30 min, characterized by quantitative ^13^C NMR with the assignment range shown in Table 2. The results are described as the percentage of carbon.

**Figure 9:**
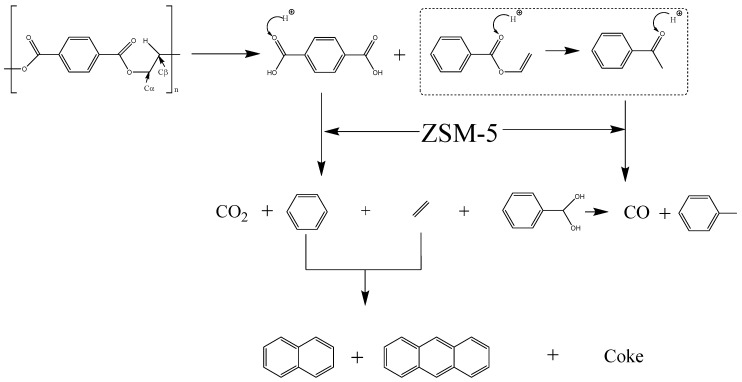
Possible decomposition pathways of PET pyrolysis.

The typical ^13^C NMR spectra for the waxy products produced by the pyrolysis of PET without any catalyst and with ZSM-5 zeolite (2.0/1.0) are shown in Figure 7. As summarized in Figure 8, the integration results of this analysis indicate that the carbonyl groups decreased significantly by 42.02% and 37.58% respectively in the wax with catalyst compared to the control. About 50% of aromatic ether are eliminated after the use of zeolite as a catalyst with the mass ratio of 1.0/2.0. The proportion of aromatic ether substituted carbons (C–O) that existed in the waxy product decreased with the addition of ZSM-5 catalyst, while this result is inconsistent with the analysis of the use of NiCl_2_. Moreover, the aromatic C–C bonds increased the use of the catalyst. This observation agreed well with the results suggested by Du et al. [13], who reported a five-fold and 10-fold increase of benzene and benzene derivatives respectively after the use of ZSM-5. Those results show that the zeolite catalyst could facilitate the cleavage of ether bonds in pyrolysis vapors. Hence, it can be concluded that ZSM-5 zeolite could promote the pyrolysis of the majority of oxygen-containing including carbonyl (C=O), aliphatic C–O, and aromatic C–O in the pyrolytic volatile products, which suggests that a smaller proportion of oxygen-containing groups were discovered in the waxy products remaining after the deoxidation of volatile products produced by the catalytic pyrolysis of PET with ZSM-5 zeolite as a catalyst. In addition, the proportion of aliphatic C–C bonds decreased by 29.64% from 21.22% to 14.93% in the presence of zeolite. This finding agreed with that of Al-asadi et al. [16], who reported that the concentration of n-olefins and n-paraffins decreased from around 32% to 23%. On the contrary, the percentage of aromatic C–C bonds were found to have an obvious increase after the addition of ZSM-5 zeolite. It seems to confirm that ZSM-5 enhances all the deoxygenation reactions suggested by Du et al. [13]. A possible decomposition route of ether bonds in PET is demonstrated as shown in Figure 9 [14]. The primary thermal cracking of PET experiences a periodic hydrogen transition process, where the hydrogen connected to Cβ transfers to the oxygen atom located at the C=O bond, followed by the consecutive scission of the alkoxy Cα–O bond and Cβ–H bond, which caused the formation of the C=C bond and O–H bond [10]. Acetaldehyde and ethylene were produced from a McLaffery rearrangement of vinyl benzoate, theoretically [31]. However, aldehyde and vinyl were barely found in the waxy product during the pyrolysis of PET with/without catalyst at any conditions, which is consistent with the results of the previous literature [13,32,36]. In addition, the C=O bond are also attacked by external protons in BrØnsted acid sites, leading to the formation of benzene free radicals and carbon oxides. Thilakaratne et al. have suggested that benzene free radicals could react with olefins to produce naphthalenes [37]. The analysis of pyrolysis residue and gaseous products in the previous literature proved that the solid residue is mainly composed of polycyclic aromatic hydrocarbons (PAHs) [13], and carbon oxides (CO_2_, CO) [12,15] account for a large proportion of gases. The same results can be concluded in our work, and it explains the high yield of carbon oxides in catalytic pyrolysis. Therefore, the major pyrolysis products were proved to be terephthalic acid and benzoic acid vinyl ester in the initial stage during the pyrolysis of PET, which agreed well with the studies of Grause et al. [10]. The ester was further transformed into acetophenone owing to the instability of benzoic acid vinyl [13,38]. ZSM-5 zeolite has an efficient deoxygenation ability for oxygen-containing groups such as carboxylic and ketone groups [39,40]. Based on the proposed pathways, the results of ^13^C NMR characterization indicate that the conversion of carbonyl groups to aromatic hydrocarbon was enhanced because of the presence of ZSM-5 zeolite during the pyrolysis of PET. For the three pyrolytic experiments of PET with different dosages of ZSM-5, the pyrolysis experiments with 2.0/1.0 (W_ZSM-5_/W_PET_) zeolite was proved to have better upgrading effects than others.

On the other hand, a complete decrease of aliphatic C–O was found in the waxy product after the use of nickel chloride as the catalyst. There are more aromatic C–C bonds and less aliphatic C–C bonds with the increasing catalyst/PET mass ratio. However, the CH_3_–Ar groups are mostly eliminated after pyrolysis with nickel chloride as the catalyst. Nickel chloride promotes the decomposition of alkyl, alkoxy, and branched alkanes on the aromatic ring. This is consistent with the previous study of Zou et al. [5].

## 4. Conclusions

The pyrolysis of PET with different dosages of ZSM-5 zeolite and NiCl_2_ as a catalyst was carried out at different temperature (450–600 °C) under N_2_ atmosphere. The yield of pyrolysis products produced from PET indicates that both the mass ratio of the catalyst to PET and temperature play a significant role in the distribution of products. The addition of ZSM-5 and NiCl_2_ reduced the yield of the waxy product but increased the yield of gas. At low temperature (450 °C), the solid residue content was almost the same level after the use of ZSM-5 as the catalyst, while the yield of char sharply decreased by using NiCl_2_ as the catalyst. In contrast, it shows an analogous trend under high-temperature conditions (600 °C). The results suggest that ZSM-5 has little effect on the primary decomposition of PET, but it promotes the secondary volatile reactions. The use of NiCl_2_ as a catalyst will greatly improve the primary decomposition of PET to generate more liquid products. In addition, the detailed analysis of ^13^C NMR spectrum for the waxy products indicates that the carbonyl groups and the aliphatic C–O bonds are effectively removed by 42% and 20% respectively after the use of ZSM-5 as a catalyst compared to the pyrolysis of PET without a catalyst. In addition, the results of integration show that the waxy products have a relatively lower oxygen content in the presence of ZSM-5, which indicates that ZSM-5 could also promote the deoxygenation during the pyrolysis of PET.

## Figures and Tables

**Table 1:** Proximate and ultimate analysis of poly (ethylene terephthalate) (PET).

**Proximate Analysis** **(wt % dry basis)**	**Volatiles**	**Fixed Carbon ^a^**	**Ash**
	88.54	9.37	2.09
**Ultimate Analysis** **(wt % dry basis)**	C	H	O ^a^
	61.87	4.35	33.78

^a^ By difference.

**Table 2:** The ^13^C nuclear magnetic resonance (NMR) chemical shift assignment ranges and functional groups distributions of PET pyrolysis oil based on the chemical shift database created by ben et al. [29].

Functional Group	-	Integration Region(ppm)
Carbonyl or carboxyl bond	-	215.0–166.5
Aromatic C–O bond		166.5–142.0
Aromatic C–C bond		142.0–125.0
Aromatic C–H bond		125.0–95.8
Aliphatic C–O bond		95.8–60.8
Methoxyl-aromatic bond		60.8–55.2
Aliphatic C–C bond	general methyl-aromatic (CH_3_–Ar) methyl-aromatic at ortho position of a hydroxyl or methoxyl group (CH_3_–Ar’)	55.2–0.0 21.6–19.1 16.1–15.4
